# Photo-induced reduction of graphene oxide coating on optical waveguide and consequent optical intermodulation

**DOI:** 10.1038/srep23813

**Published:** 2016-04-01

**Authors:** W. Y. Chong, W. H. Lim, Y. K. Yap, C. K. Lai, R. M. De La Rue, H. Ahmad

**Affiliations:** 1 Photonics Research Centre, Physics Department, Science Faculty, University of Malaya, 50603 Kuala Lumpur, Malaysia; 2 Optoelectronics Research Group, School of Engineering, University of Glasgow, Glasgow G12 8QQ, United Kingdom; 3 Heriot-Watt University Malaysia, Putrajaya, 62200 Federal Territory, Malaysia

## Abstract

Increased absorption of transverse-magnetic (TM) - polarised light by a graphene-oxide (GO) coated polymer waveguide has been observed in the presence of transverse-electric (TE) - polarised light. The GO-coated waveguide exhibits very strong photo-absorption of TE-polarised light - and acts as a TM-pass waveguide polariser. The absorbed TE-polarised light causes a significant temperature increase in the GO film and induces thermal reduction of the GO, resulting in an increase in optical-frequency conductivity and consequently increased optical propagation loss. This behaviour in a GO-coated waveguide gives the action of an inverted optical switch/modulator. By varying the incident TE-polarised light power, a maximum modulation efficiency of 72% was measured, with application of an incident optical power level of 57 mW. The GO-coated waveguide was able to respond clearly to modulated TE-polarised light with a pulse duration of as little as 100 μs. In addition, no wavelength dependence was observed in the response of either the modulation (TE-polarised light) or the signal (TM-polarised light).

Waveguide-based optical modulators and switches are essential devices in optical communications for functionality such as on-chip interconnection, optical signal processing, integrated optoelectronic circuits, network protection and as the fundamental building-blocks of optical logic gates^1^,^2^,^3^,^4^. All-optical modulation has unique advantages in optical signal processing compared with modulation based on electro-optic or acousto-optic effects. To date, many all-optical switching approaches have been reported and developed, and have shown good modulation depth with demonstrated high speed response^5^,^6^,^7^,^8^. Most, if not all, reported optical modulators have an intrinsically narrow operating wavelength range and therefore require sophisticated design for applications that involve wavelength independence. An alternative approach for broadband all-optical modulation is the use of materials that possess an intrinsically broadband response^9^.

Graphene photonics has become an important research field, due to the unique characteristics of graphene - which extend to optical frequencies^10^,^11^. Graphene based devices such as broadband polarisers and modulators have been developed^10^,^12^,^13^,^14^. The broadband response of graphene is a result of the fact that its conductance is independent of frequency over a wide range^11^,^12^,^13^,^14^,^15^. Moreover, the strong light-graphene interaction and high carrier mobility properties of graphene are additional advantages for optical modulation applications. Sharing similar advantages to those of graphene - while at the same time also possessing its own unique properties - graphene oxide (GO) has been used as a functional element in both passive and active photonics devices that include mode-locked fibre lasers and waveguide polarisers^16^,^17^. The polariser was realised by exploiting the ability of GO to provide large-area uniform coating, using a drop-casting technique and the strongly anisotropic complex dielectric function of multilayer GO^18^,^19^. However, understanding of the optical characteristics of GO still needs to be strengthened and the characteristics compared with those of graphene. Particularly, the strong polarisation-selective absorption of GO films is expected to have an effect on the light propagation characteristics of GO-coated optical waveguides.

In the present study, the effects of the absorption of TE-polarised light by multilayer GO films on the optical characteristics were studied using evanescent field coupling. Increased attenuation of the TM-polarised light was observed when the power level of the incident TE-polarised light was significantly increased. A potentially useful inverted modulation depth of 72% (limited by the maximum modulation power level) was measured in the 1520 to 1590 nm wavelength range (limited by the available optical bandwidth of the probe source). The modulation effect is identified as being due to reversible photothermal reduction of the GO film, with the thermal energy being generated by strong TE-polarised light absorption in the GO film.

## Experimental Procedure

### Preparation of GO-coated optical waveguide

SU-8 polymer waveguides were fabricated on silicon substrates with Benzocyclobutene (BCB) being used as the under-cladding layer. A 6 μm thick BCB layer was spin-coated onto a silicon substrate - and then a 3 μm thick SU-8 layer was spin-coated onto the BCB layer. The refractive indices of BCB and SU-8 are 1.540 and 1.579 respectively, measured at 1550 nm using a prism coupler apparatus (Sairon SPA-4000). The sample then underwent photolithography and subsequent chemical processing to produce straight channel waveguides with a nominal width of 3 μm. GO solution, prepared using an improved Hummer’s method and dispersed in water, was then drop-cast onto the channel waveguides. The concentration of the GO solution was 1 μg/μL and the droplet volume used was 1 μL. The GO solution was allowed to dry at room temperature for thirty minutes. Optical resin (NOA 68) with a refractive index of 1.51 was applied to the entire channel waveguide to avoid direct exposure of the channel waveguide to the external environment. The GO-coated waveguide showed very strong polarisation selective transmission characteristics, as explained in detail in [17]. The thickness of the GO film coating was controlled by the number of solution droplets applied during drop-casting of the GO solution. In this case, two drops of GO solution were used to produce a total GO film thickness of approximately 0.75 μm.

### Optical test and measurements

**Figure 1:**
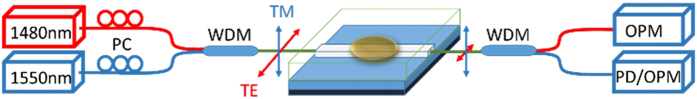
Experimental setup for optical switching measurement. PD – Photodiode; OPM – Optical power meter; WDM – Wavelength Division Multiplexer; PC – Polarisation controller.

The experimental setup for optical switching measurement is depicted in Fig. 1. High numerical aperture fibres (UHNA-4) were used to butt-couple to the GO-coated waveguide via a pair of 5-axis fibre alignment stages. The total fibre-to-fibre insertion loss of the GO-coated waveguide was ~7.4 dB, with the main contributing factors being the refractive-index magnitude mismatch and the modal-profile mismatch - and the corresponding NA mismatch between the coupling fibres and the optical waveguide. Two separate laser sources were used as the optical control and the optical signal. The control-laser was a Fabry-Perot laser diode operating at 1480 nm (Sumitomo SLA5600), while the signal-laser was a tunable laser source operated at 1550 nm (Santec ECL-210). A broadband 1550 nm superluminescent diode (SLD) source (Acterna OBS-15) was also used as the signal source to measure the wavelength response of the GO-coated waveguide in the 1550 nm wavelength band. The control and signal sources were combined using a 1480/1550 wavelength division multiplexer (WDM) device, while a second WDM device with the same filtering characteristics was used to separate the two laser wavelengths at the output, for convenience of analysis. Polarisation controllers were placed between the laser sources and the input of the WDM device, in order to allow control over the polarisation state of both laser sources before they were coupled into the GO-coated waveguide. It is worth noting that the WDM device did not have any polarisation-dependent loss. The output power levels at both wavelengths were analysed using an optical power meter (ILX Lightwave OMM-6810B), while the spectrum of the output was measured using an optical spectrum analyser (Yokogawa AQ6370). A polarisation analyser (Thorlabs PAN5710IR3) was used to analyse the polarisation state at both wavelengths and the modulation speed was measured using a fast photodetector (Thorlabs DET-36). To measure the temperature of the GO film, a GO-coated waveguide was fabricated with the GO-coated region left uncovered. A mini-hypodermic T-type thermocouple (HYP-0, Omega) was carefully placed at a distance of less than 1 mm from the GO-coated waveguide near the input end - and in physical contact with the GO film. An optical waveguide without any GO coating was also fabricated as a reference sample. Reproducibility of the modulation effect was verified by comparing the modulation performance of three separate GO-coated waveguides.

## Results and Discussion

**Figure 2:**
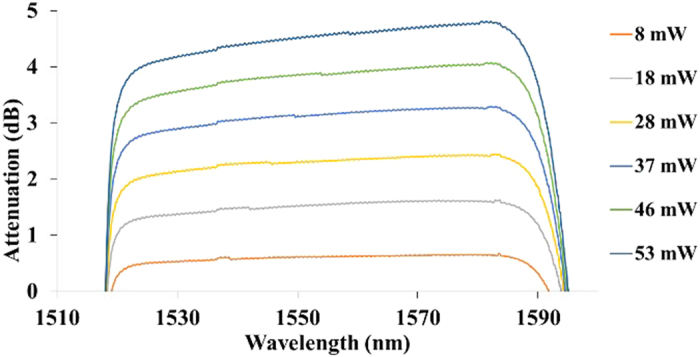
Broadband attenuation of the signal-source power by the optical switch at different CW control–source power levels. The measurement range between 1525–1580 nm was limited by the transmission window of the WDM device used.

Figure 2 shows the attenuation of the 1550 nm superluminescent diode emission in the TM-polarisation state by the GO-coated waveguide - at different control-source power levels. The SLD output is highly polarised and hence control of its polarisation state is straightforward - using the polarisation controller. The 1480 nm control-source was operated in continuous-wave mode and was TE-polarised. The measurement wavelength range was limited to 1525–580 nm by the transmission window of the WDM device used. It was observed that the attenuation of the SLD spectrum increased as the control-source power level was increased. Attenuation of the SLD spectrum was observed over the entire available transmission spectrum from 1525 nm to 1580 nm, indicating the spectrally broadband response of the optical switch. Figure 2 shows only the static spectrum, with no time factor being available for analysis. The 1480 nm control-source was set to a fixed power level - and the output from the waveguide switch was measured using an optical spectrum analyser. For each value, multiple scans were performed to ensure that the spectrum was reproducible and stable. The time taken for a single scan was about 30 seconds.

When the reference sample (optical waveguide without GO coating) was tested, the same effect was not observed. Instead, a small increase in the power level was measured from the signal-source WDM device output, due to the imperfection of the WDM filtering at a high control-source power level. On the other hand, when the polarisation of the control-source was changed from TE-mode to TM-mode, no suppression effect was observed. Since TM-polarised light is less absorptive in multi-layer GO films, the two observations above indicate that the suppression effect is caused by the absorption of TE-polarised light by the GO film. In addition, when the polarisation of the signal-source was changed to give TE-mode guiding, the transmitted power was always low - and the suppression effect, if any, was not observable.

**Figure 3:**
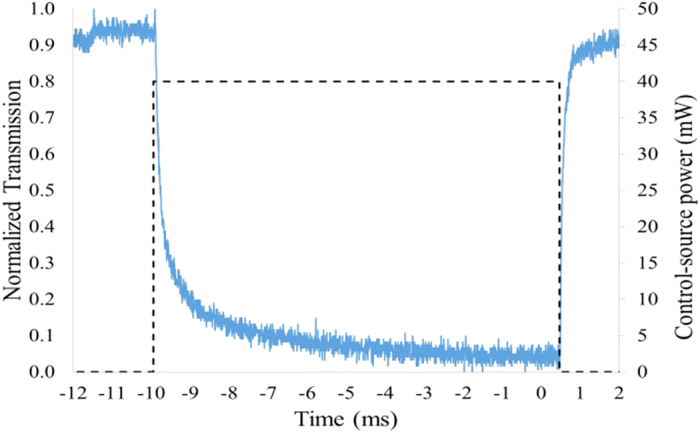
Response time of the optical switch when a 10 ms control-source square optical pulse is introduced.

The response time of the optical switch was also examined. The SLD was replaced with a single wavelength 1550 nm laser source to provide a signal-source with a higher polarisation extinction ratio and power level stability. Figure 3 shows the response of the optical switch when a 10 ms control-source optical pulse was introduced. The time taken for the normalized transmission to fall to the 30% level was estimated to be approximately 350 μs - while the time taken for the normalized transmission to recover to the 80% level was approximately 250 μs.

**Figure 4:**
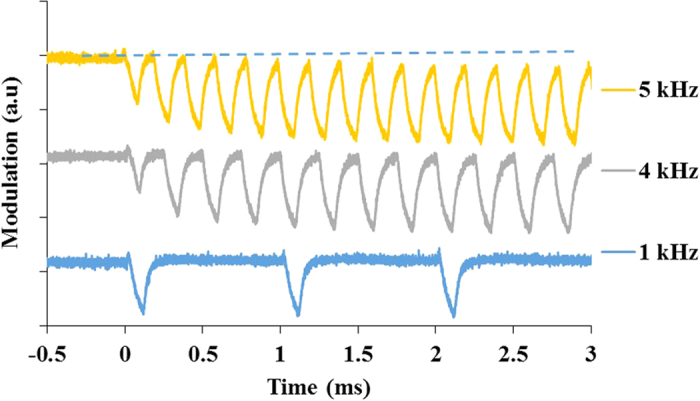
Modulation depth of the optical switch measured using a photodiode at 3 different modulation frequencies with fixed pulse durations of 100 μs. The traces for 4 kHz and 5 kHz have been shifted up for easy viewing.

The response and the modulation efficiency of the optical switch were further investigated using higher modulation frequencies. Figure 4 shows the time-dependent variation of the signal-source power level, measured using a photodiode, when the control-source was modulated at three different frequencies, but at a fixed pulse duration of 100 μs - and limited by the 1480 nm control-source laser. The control-source modulation starts at 0 s. It can be seen that the optical switch is able to respond to the set optical pulses at these repetition frequencies. For a modulation frequency of 1 kHz, the pulse-to-pulse modulation depth was stable and no significant changes in the maximum and minimum power levels were observed. When higher modulation frequencies (4 kHz and 5 kHz) were used, a drop in the maximum power level was observed over time, and this power drop resulted in a progressively lower modulation depth - before the situation stabilised after about 2 ms.

**Figure 5:**
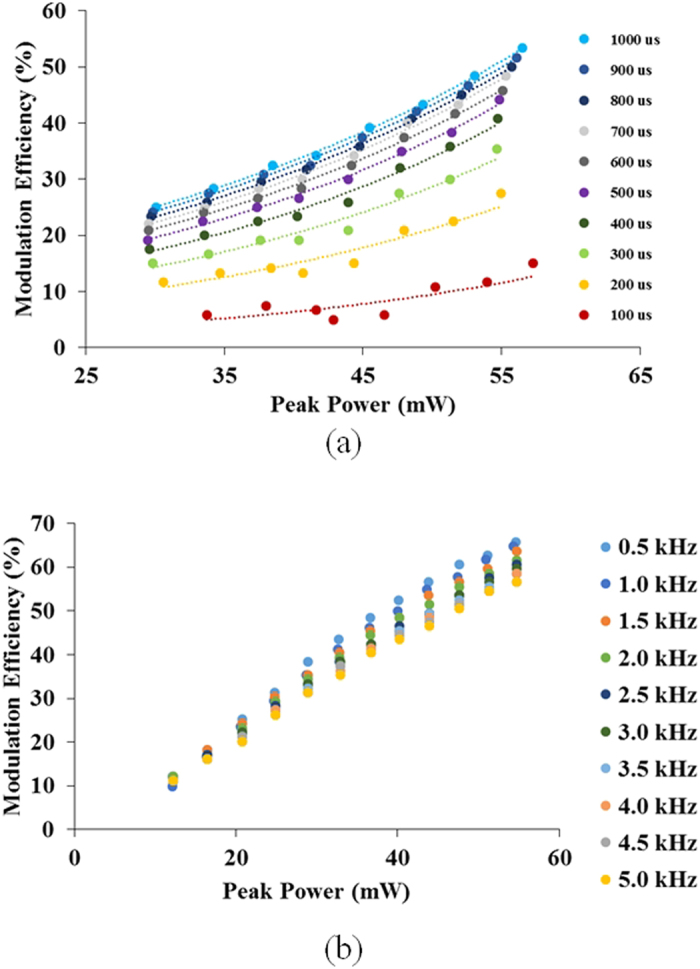
(**a**) Modulation efficiency of the switch at various pulse durations, modulated at 50 Hz. The dotted line was included for easy viewing. (**b**) Modulation efficiency of the switch for modulation frequency in the range from 0.5 kHz to 5 kHz with fixed 50% duty cycle.

The modulation efficiency was studied by modulating the control-source at different pulse repetition frequencies, power levels and pulse durations. Figure 5(a) shows the modulation efficiency of the optical switch at a low modulation frequency (50 Hz) - and with the pulse duration varied over the range from 100 μs to 1000 μs. When modulated with short, approximately rectangular, optical pulses, a slight increase in the modulation efficiency of the optical switch with control-source peak power was measured. The modulation efficiency was also relatively low, with a maximum efficiency of 15% when the control-source power level was set at 57 mW. As the pulse duration increased, so did the modulation efficiency. At the same time, the gradient of the modulation efficiency with the peak power level of the control-source also increased. The optical switching shows a linear response to control-source power, indicating that the suppression effect observed is not likely to be due to non-linear effects in the GO film.

The optical switching performance was also studied at higher modulation rates. Figure 5(b) shows the modulation efficiency of optical switching for modulation frequencies ranging from 0.5 kHz to 5 kHz, at a fixed duty cycle of 50%. The linear response of the modulation efficiency to the control-source power level appears to be retained. However, while there is almost no difference in the modulation efficiency for the different modulation frequencies at low control-source power levels, the modulation efficiency appeared to be frequency dependent when greater control-source power levels were used. Lower modulation efficiency was observed for higher modulation frequency. This result leads to the conclusion that the optical power level reduction mechanism in the multilayer GO film is related to thermal effects.

**Figure 6:**
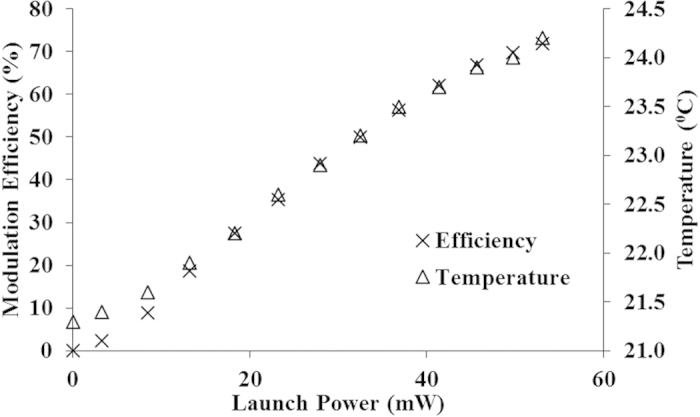
Modulation efficiency at various control laser power levels and corresponding temperature change of GO coating measured near the GO-coated optical waveguide.

**Figure 7:**
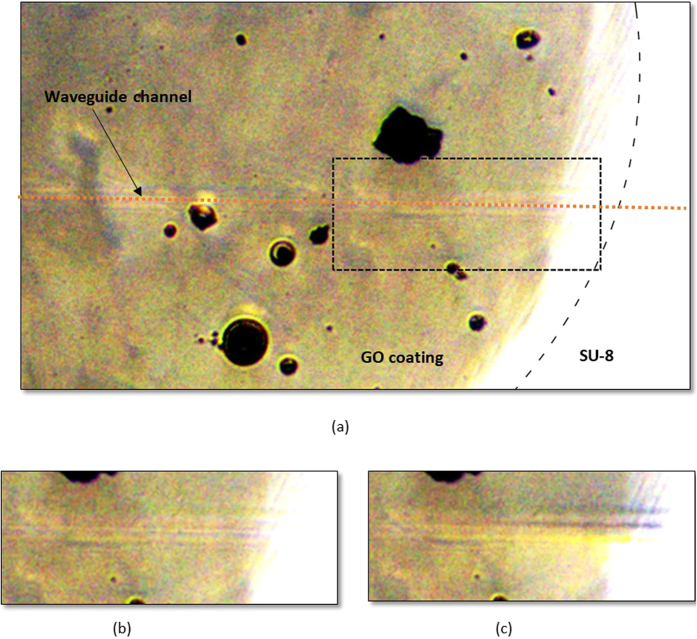
(**a**) Top view of GO-coated waveguide with magnification of the rectangle region shown in (**b**) when no TE-polarised light is coupled into the waveguide and (**c**) when TE-polarised light is coupled into the waveguide and causes darkening of the GO film above the waveguide channel. Contrast level has been adjusted to enhance the darkening effect.

To verify the above hypothesis, the variation in the modulation efficiency of the optical switch with the continuous wave (CW) control-source power level was measured - and the result is shown in Fig. 6. A modulation efficiency of 72% (limited by the maximum control-source power level) has been achieved with 57 mW of control-source power and a 1 mW signal-source power level. (It was observed that the modulation efficiency is independent of the signal-source power level). At the same time, the temperature of the GO film in a GO-coated waveguide without top cover was measured - and the temperature change for a varying control-source power level was also plotted in Fig. 6. The temperature of the GO film increased linearly with increasing control-source power, with a maximum change of 3 °C when the CW control-source power was set at 57 mW. Note that the measured temperature shares the same trend with the modulation efficiency. A relatively fast change in the GO film temperature was observed when the control-source power level was varied. It seems likely that the absolute temperature of the GO film in contact with the SU-8 waveguides is significantly higher than the measured temperature, possibly in the range of 100–200 °C - where onset of thermal reduction of GO film is known to occur. In addition, the top view image of the GO-coated waveguide with and without the presence of control-source power is shown in Fig. 7. Darkening of the GO layer covering the waveguide channel in the presence of control-source power is observed in Fig. 7(c), as compared with the GO layer when the control-source power is turned off. The darkening effect is reversible. The repeated turning on and off of the control-source power will cause the GO layer above the waveguide channel to darken and vice versa.

**Figure 8:**
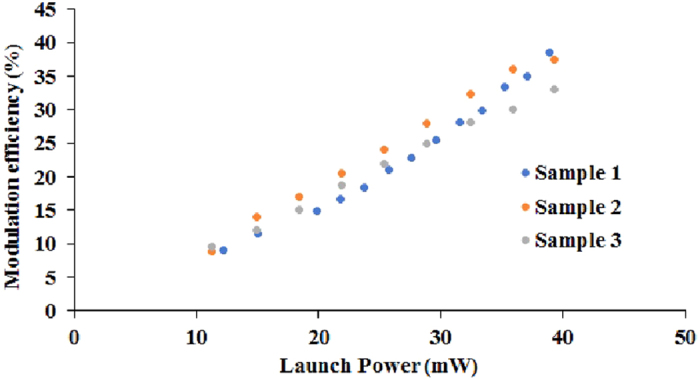
Comparison of modulation response of 3GO-coated waveguides fabricated using the same parameters.

In order to check the reproducibility of the modulation effect, the modulation response of three GO-coated waveguides was compared and the results are shown in Fig. 8. It can be seen from the figure that the modulation efficiency for all samples increases approximately linearly with the control-source power. The variation in modulation efficiency between the samples is ~10%. It should be noted that control of the modulation strength of the samples generated in different fabrication cycles has still to be improved. This issue arises mainly from technical difficulties, namely thickness variation of the GO coating deposited using the current method, which in turn affects the TE-polarised light absorption by the GO-coated waveguide, the coupling loss between different waveguides, etc. Improvement of the GO-coating process, together with use of a fully consistent and reproducible waveguide fabrication process will improve the reproducibility of the modulation response. Nevertheless, the modulation effect observed is repeatable and reproducible within acceptable limits, when using the current material and fabrication platform.

As is well known, carbon-based materials have a significant capability for self-heating through optical phonon excitation^20^,^21^,^22^,^23^. Guo^24^ has shown that a 1 μm thick GO film can undergo a significant rise in temperature (>40 °C difference, as compared with a bare substrate without a covering GO film) - when exposed to a broadband infrared light intensity of only 0.32 Wcm^−2^ (with an emission spectrum covering the range from 1000 to 1700 nm). Thermally heated GO films will undergo a de-oxygenation reaction and the GO film will therefore be substantially reduced^20^,^24^,^25^,^26^. A suitably reduced-GO film can have remarkably greater conductivity than fully oxidised GO - and eventually, therefore, much enhanced optical absorption may be observed^27^,^28^,^29^. In our optical switch, the control-laser was launched in the TE-mode - and this light was then absorbed by the GO film, producing a photo-thermal effect. The heated GO-film then had greater attenuation at the signal-laser source wavelength and eventually a transmitted light suppression effect was induced. Thermally reduced-GO film is unstable and it readily oxidises back to its initial state^24^,^30^, in an oxygen-containing ambient. This re-oxidation is especially apparent in our optical switch, where the GO film is shielded by the NOA overcladding. The oxygen molecules that are released during the photo-thermal process cannot escape because they are trapped by the NOA overcladding - and are eventually chemically bonded back into the GO layers, causing reversible changes in the suppression effect when the control-laser is turned off. While a thermally induced reaction between the multi-layer GO film and the adjacent polymer waveguide core is a possibility, it would almost certainly require the rapid partial reduction of the GO multi-layer film to occur in the observed timescale, with the same end-result of modifying the absorption of the GO film. Uniform reduction of the GO multi-layer is not required to obtain the observed modification of the waveguide propagation loss – and the GO layers closest to the interface with the polymer waveguide core are likely to be the most strongly modified, because of the optical mode-distribution.

This suppression phenomenon is not observed when light from the control-laser is launched in the TM–mode, due to the fact that the absorption cross-section of GO films for light in the TM-mode is much lower when compared with the absorption for the TE–mode - and hence a smaller photo-thermal effect occurs. Similar reasoning applies for signal-laser light that is launched in the TM–mode, which has no significant effect on suppression. It is worth noting that the control-laser can be at another wavelength, due to the broadband optical characteristics of GO. For example, when a 980 nm light source was used as the control signal, a similar outcome to that described above was obtained - a suppression effect with ~100 μs response time, but the modulation depth was much lower. The difference in the modulation efficiency between the two different situations is due to the change in light coupling into the GO film with changing wavelength. The GO film in the optical waveguide switch can itself be considered as another waveguide core and the amount of light that is guided in the GO film will depend on the GO multi-layer film thickness, as well as the wavelength. Hence the 980 nm control-laser source has a lower coupling ratio, which reduces the suppression effect. Another example occurred when the control-laser and signal laser were swapped, with the 1550 nm wavelength laser being used as the control. A similar suppression phenomenon with almost equal response time was observed. The response time observed is determined by the photo-thermal effect, regardless of the wavelength of the control-laser. So long as the environmental situation of the GO films remains unaltered, the response and relaxation times of the GO film should remain the same.

The cross-polarisation modulation effect that we have observed in a GO-coated optical waveguide is interesting from a physical chemistry view point, as well as on a device performance basis. In addition, the behaviour observed could affect the stability of optical devices that are realised by using mono- or multi-layer graphene-oxide films at various levels of reduction, including fully reduced graphene. An obvious implication is the need for effective hermetic packaging to reduce the possible impact of unintentional oxidation and/or water (vapour) adsorption. On the other hand, the optical switching performance that we have demonstrated still leaves considerable room for improvement, particularly in achieving a higher modulation efficiency and faster response times. Optimisation of the GO coating thickness and length, as well as using a thinner overcladding, to increase the rate of heat dissipation are possible approaches to improvement upon the switching performance so far demonstrated.

## Conclusions

Increased optical attenuation of TM-polarised light propagating through a GO-coated waveguide in the presence of TE-polarised light has been observed. This behaviour gives the action of an inverted optical switch/modulator. A modulation efficiency of 72% was obtained at a control-source power level of 57 mW - and the optical switch was able to respond to modulated TE-polarised light with a pulse duration of 100 μs. The cross-polarisation modulation effect that we have observed is the result of reversible photo-thermal reduction of the GO film by strong absorption of TE-polarised light. Deeper understanding of the detailed mechanisms of photo-thermal reduction – and especially its effect on graphene-based photonic devices - is needed to allow reliable and predictable utilization or mitigation of this effect. Work towards optimization of the waveguide geometry and modal distributions in the optical switch should also allow significant improvements in the switching performance. The inverted modulation characteristics that we have observed in waveguides have potential applications in optical signal processing and network protection, as well as providing a possible fundamental building block for optical logic gates.

## Additional Information

**How to cite this article**: Chong, W. Y. *et al.* Photo-induced reduction of graphene oxide coating on optical waveguide and consequent optical intermodulation. *Sci. Rep.* **6**, 23813; doi: 10.1038/srep23813 (2016).
